# Management of Periapical Lesion Associated With Root Fracture in the Elderly: A Case Report

**DOI:** 10.7759/cureus.25316

**Published:** 2022-05-25

**Authors:** Geraldo Matos

**Affiliations:** 1 Dentistry, Faculdade de Medicina de São José do Rio Preto (FAMERP), São José do Rio Preto, BRA

**Keywords:** treatment, dental trauma, periapical lesion, root fracture treatment, root fracture

## Abstract

The early detection and management of root fractures pose a challenge to dentists. There are two treatment options in such cases: conservative and endodontic or surgical. Follow-up of such cases involves the continual evaluation of the tissue repair process and the possible occurrence of undesirable events, such as periapical lesions, pulp calcification, and internal or external resorption. This case report describes the finding of a periapex with relatively extensive bone rarefaction resulting from a root fracture in a 64-year-old male who presented with gingival hyperplasia in the right maxillary lateral incisor region sensitivity at the touch of the exploratory probe and mild bleeding with exudate. In this case, the early diagnosis of root fracture enabled the choice of the best therapeutic option with a satisfactory outcome.

## Introduction

The early detection and management of root fractures pose a challenge to dentists. Root fractures involve the cementum, dentine, pulp, and periodontal ligament and can occur in the cervical, middle or apical third, with or without mobility and with or without displacement [1]. The prevalence of root fracture in permanent teeth ranges from 0.5 to 7.7% [2,3]. Moreover, root fractures occur mainly in the central incisors because these teeth are in the most prominent position in the dental arch [4,5].

The two treatment options for root fracture are conservative and endodontic or surgical. Follow-up of such cases involves the continual evaluation of the tissue repair process and the possible occurrence of undesirable events, such as periapical lesions, pulp calcification, and internal or external resorption. Thus, it is essential to maintain patients in clinical and radiographic follow-up [6]. This paper reports a clinical case of an elderly patient treated for a periapical lesion associated with a root fracture.

## Case presentation

**Figure 1:**
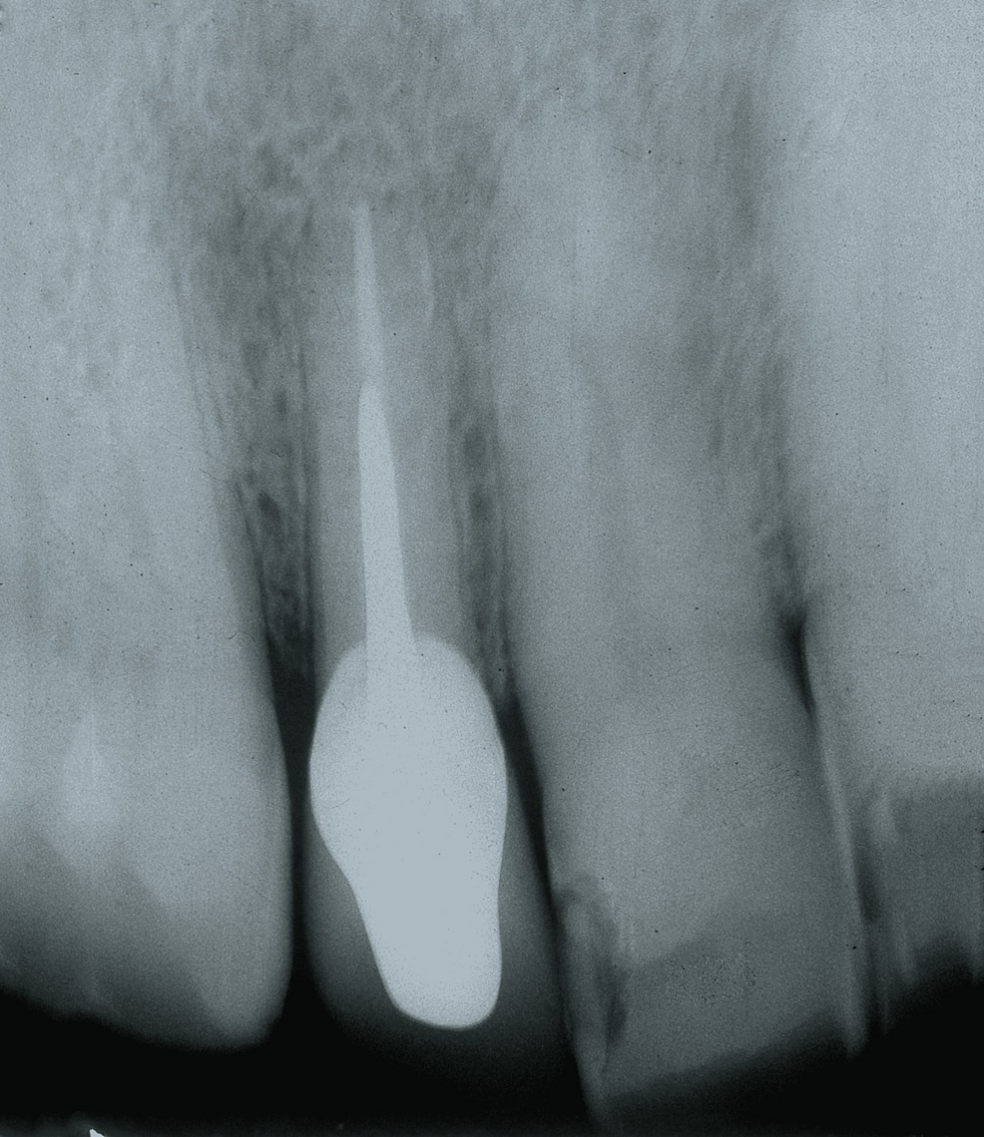
Periapical radiographic images showing root fracture

**Figure 2:**
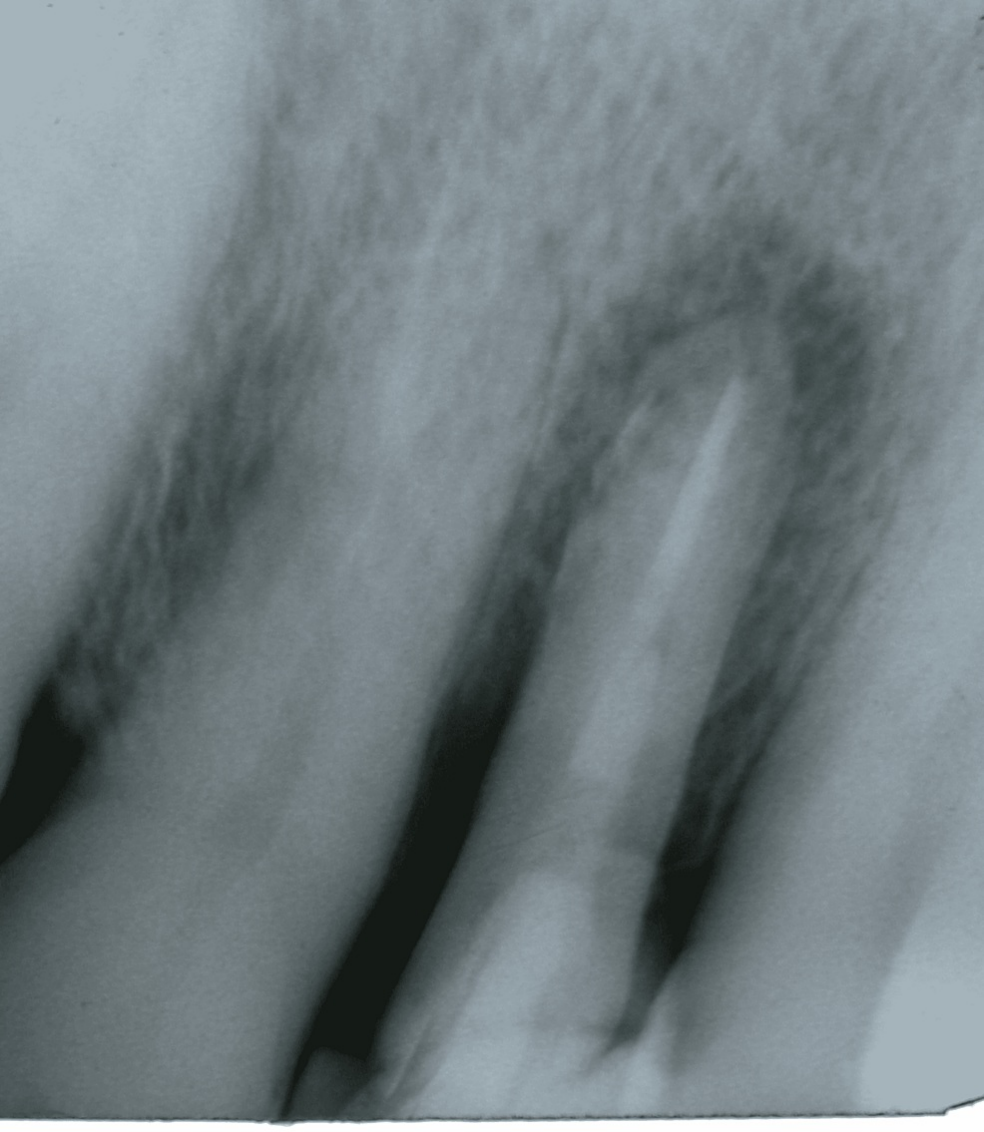
Periapical radiographic images showing a radiolucent area of apex with bone rarefaction on Tooth 12

A 64-year-old male sought dental care. The intraoral clinical examination showed gingival hyperplasia in the region of the right maxillary lateral incisor (Tooth 12). The patient reported sensitivity to the touch of the exploratory probe, and mild bleeding with exudate was observed. The periapical x-ray revealed a radiolucent image of the periapex with relatively extensive bone rarefaction resulting from a root fracture (Figures 1, 2). Extraction of the tooth was indicated.

For the surgical procedure, antibiotic therapy was prescribed with Amoxycillin and potassium clavulanate (875 mg + 125 mg, two pills per day for one week). The pre-anesthetic was 200 mg/g of topical benzocaine (Benzotop, Nova DFL, Rio de Janeiro, RJ, Brazil), followed by the local anesthetic of phenylephrine lidocaine (S.S. White 100, Rio de Janeiro, RJ, Brazil). The nucleus, crown, and remaining root were surgically removed, followed by curettage irrigated with saline solution. The suture was performed with 4-0 Shalon® silk (Shalon Fios Cirúrgicos Ltda, Goiânia, GO, Brazil). The following day, the temporary tooth was placed with an adhesive system for enamel and dentine (Ambar 6 mL, FGM, Joinville, SC, Brazil) using Z-250 polymerizable composite resin (3M, Sumaré, SP, Brazil).

**Figure 3:**
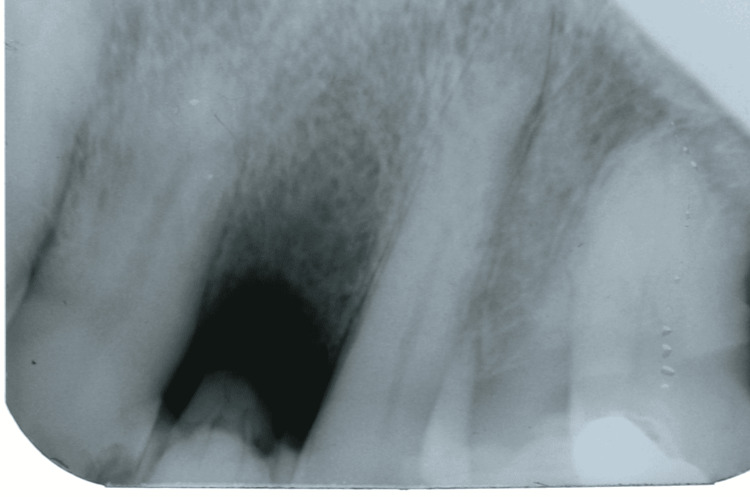
Periapical radiographic image showing onset of bone formation after six months

**Figure 4:**
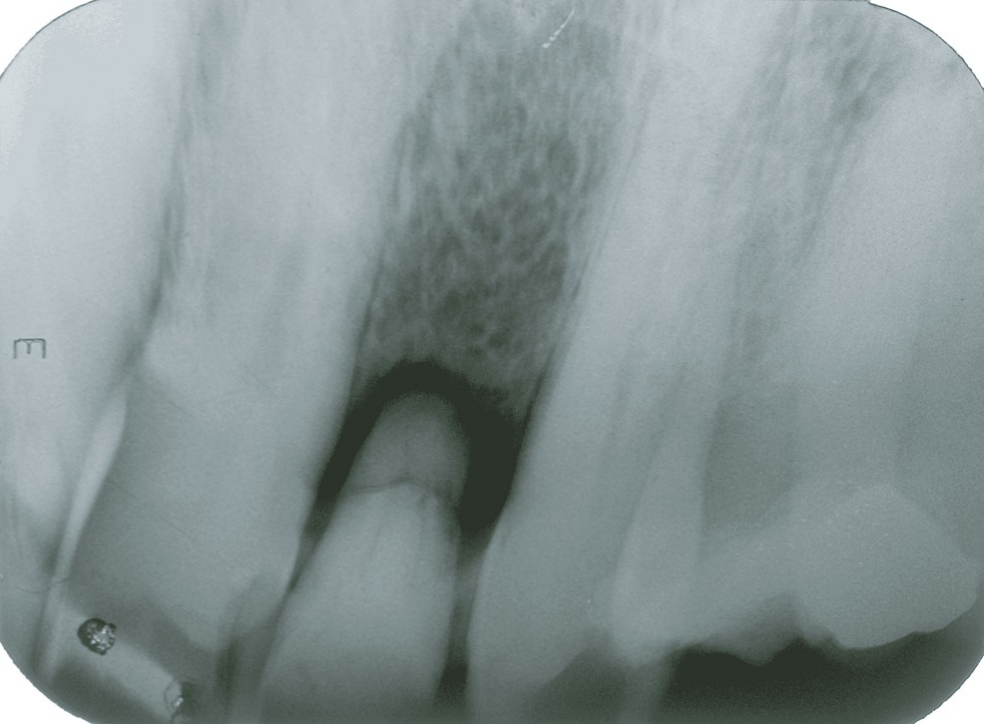
Periapical radiographic image showing bone formation after eight months

**Figure 5:**
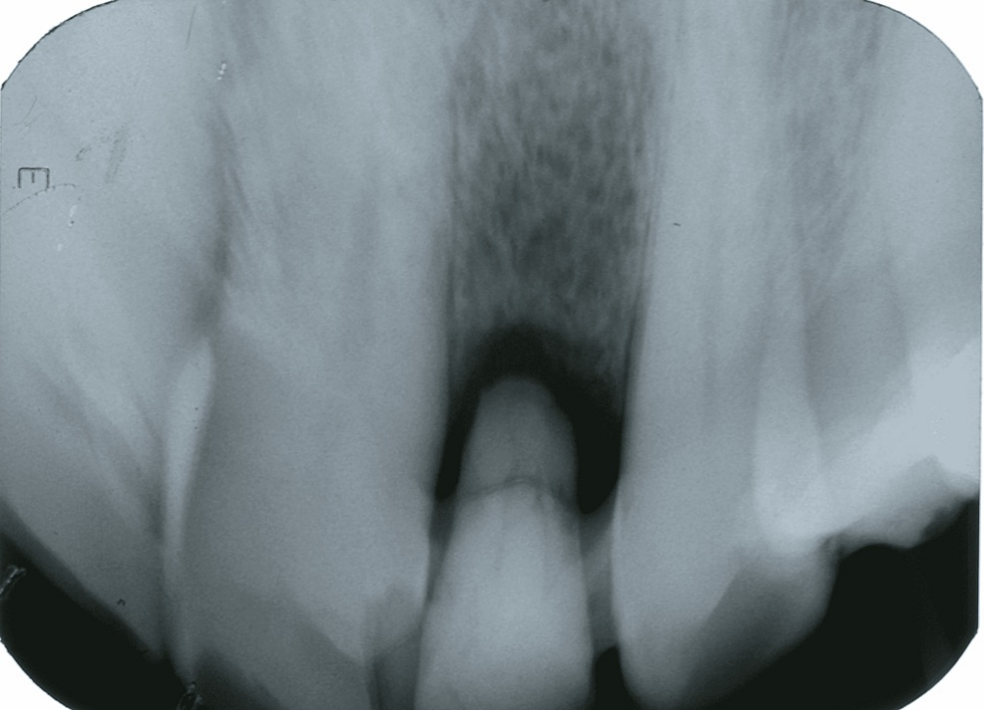
Periapical radiographic image showing formed bone tissue after 12 months, with an indication for dental implant

**Figure 6:**
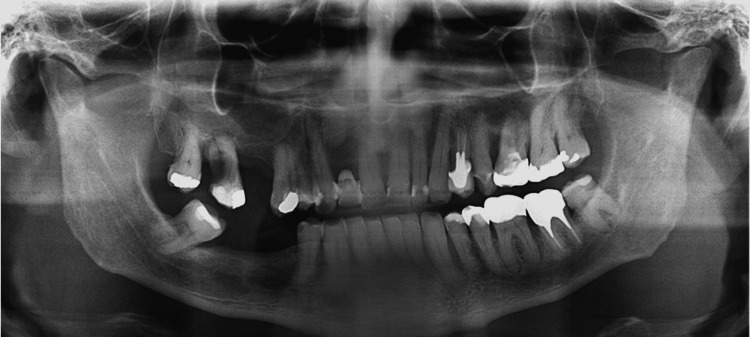
Radiographic panoramic image showing formed bone tissue after 12 months, with an indication for dental implant

At the six-month follow-up, the periapical x-ray revealed a reduction in bone rarefaction in the region of the right maxillary lateral incisor (Figure 3). Bone formation was found at the eight-month follow-up (Figure 4), indicating a satisfactory response to treatment. At 12 months, formed bone tissue was found, and a dental implant was indicated (Figures 5, 6).

## Discussion

Traumatic dental injuries are recognized as a public dental health problem worldwide, and these injuries are more prevalent in permanent dentition [7]. According to the World Health Organization classification, these injuries include trauma to the hard dental tissues and the pulp, the periodontal tissues, the supporting bone, and gingiva and oral mucosa [8]. Among the trauma to the hard dental tissues and the pulp, the root fractures are the most commonly found emergencies in the dental clinic with social, functional, and esthetic complications [9].

Treatment for this type of fracture depends on the location of the fracture line, proximity to the gingival sulcus, tooth mobility, and the pulp tissue condition [10]. In this case, the early detection of the fractured root and extraction of the tooth was fundamental, aiming to maintain the integrity of alveolar bone for the placement of an implant.

Regarding etiologic factors for root fracture, a metal pin concentrates stress on the root due to the high modulus of elasticity, leading to a high incidence of root fractures [11]. This was confirmed in the present case, as the root fracture was associated with a tooth that had a metal pin. Furthermore, alterations to the tooth with patient age may contribute to root fractures because there is a gradual reduction in the tubule diameter with increasing age [12].

Considering that the detection of root fracture is commonly challenging for clinicians, mainly when the results from the typical clinical diagnostic tests are indecisive, the root fractures require the proper radiographic diagnosis to determine the extent and therapeutic options, along with a complete assessment of the history of the case and clinical examination of the tooth structures and bone. Moreover, the follow-up of the patients’ oral hygiene and radiographic control to detect early signs of any disease are essential in trauma cases.

In the present case, the precise diagnosis of a periapex with relatively extensive bone rarefaction resulting from a root fracture in a 64-year-old male who presented with gingival hyperplasia, sensitivity at the touch, and mild bleeding with exudate, enabled the choice of the best treatment option, which contributed to successful treatment, as demonstrated during the clinical and radiographic follow-up evaluations. Based on this context, dentists should be adequately trained and aware of the importance of a precise diagnosis in such cases.

## Conclusions

The report of this clinical case of an elderly patient shows that early diagnosis of root fracture enabled the choice of the best therapeutic option with a satisfactory outcome, as demonstrated at the six-month clinical and radiographic follow-up evaluation. When the typical clinical diagnostic tests are indecisive, the root fractures require the proper radiographic diagnosis to determine the extent and therapeutic options, along with a complete assessment of the history of the case and clinical examination of the tooth structures and bone. Dentists should be adequately trained and aware of the importance of a precise diagnosis in elderly patients with a periapical lesion resulting from a root fracture.
